# Novel adaptive beam-dependent margins for additional OAR sparing

**DOI:** 10.1088/1361-6560/aae658

**Published:** 2018-10-29

**Authors:** H S Tsang, C P Kamerling, P Ziegenhein, S Nill, U Oelfke

**Affiliations:** pmb1 Joint Department of Physics, The Institute of Cancer Research and The Royal Marsden NHS Foundation Trust, London SM2 5NG, United Kingdom; henry.tsang@icr.ac.uk

**Keywords:** radiotherapy, margins, treatment planning, adaptive margins

## Abstract

Margins are employed in radiotherapy treatment planning to mitigate the dosimetric effects of geometric uncertainties for the clinical target volume (CTV). Unfortunately, whilst the use of margins can increase the probability that sufficient dose is delivered to the CTV, it can also result in delivering high dose of radiation to surrounding organs at risk (OARs). We expand on our previous work on beam-dependent margins and propose a novel adaptive margin concept, where margins are moulded away from selected OARs for better OAR-high-dose sparing, whilst maintaining similar dose coverage probability to the CTV. This, however, comes at a cost of a larger irradiation volume, and thus can negatively impact other structures. We investigate the impact of the adaptive margin concept when applied to prostate radiotherapy treatments, and compare treatment plans generated using our beam-dependent margins without adaptation, with adaption from the rectum and with adaptation from both the rectum and bladder. Five prostate patients were used in this planning study. All plans achieved similar dose coverage probability, and were able to ensure at least 90% population coverage with the target receiving at least 95% of the prescribed dose to 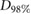. We observed overall better high-dose sparing to OARs that were considered when using the adapted beam-dependent PTVs, with the degree of sparing dependent on both the number of OARs under consideration as well as the relative position between the CTV and the OARs.

## 1. Introduction

The presence of geometrical uncertainties in external beam radiation therapy results in deviations between the planned dose distribution and the dose distribution that is physically delivered to the patient. These uncertainties arise from various sources, for example organ motion and patient set up uncertainties. If unaccounted for, these geometric uncertainties can result in underdosage of the clinical target volume (CTV), or overdose of organs at risk (OARs).

Current clinical practice account for these uncertainties using safety margins to define a planning target volume (PTV), such that sufficient coverage of the PTV equates to delivering sufficient dose to the CTV to a clinically acceptable probability. To achieve a higher probability in the CTV receiving sufficient dose coverage, a larger PTV is needed. However, the larger the irradiation volume, the more dose we are delivering to the patient, thereby increasing the probability of normal tissue toxicity.

The most widely used strategy used to determine the required size of the CTV to PTV margin is the recipe proposed by Van Herk *et al* (2000). Tsang *et al* (2017) proposed modifications to the margin concept by considering margins on a beam-by-beam basis. However, these margin concepts are typically agnostic to any other regions of interest in their formulation. In particular, these margin formulations do not consider the presence of nearby OARs and the possibility of the PTV overlapping with OARs. In the event where the PTV overlaps an OAR, the International Commission on Radiation Units and Measurements (ICRU) Report 83 (ICRU 2010) recommends either using priority rules for treatment planning optimisation (i.e. treat all or part of the overlap as the PTV or the OAR), or to subdivide the PTV into regions with different dose prescription to better spare the overlapping OAR.

Probabilistic treatment planning strategies provide alternative means to ensure sufficient target coverage probability, without the need for defining a PTV in treatment planning, by incorporating the uncertainties directly into the inverse-planning optimisation process. As the treatment planning process no longer pre-define the extent of the high-dose region by planning to the PTV, but is planned to the CTV with the geometric uncertainties as additional parameters, the presence and dose limits of the OARs can be considered as well. Examples of such techniques include coverage optimised planning (Gordon *et al* 2010, Xu *et al* 2014, Mescher *et al* 2017), expected percentile dose coverage optimisation (Tilly *et al* 2017), and Witte *et al* (2017) exploring the extent of the high dose region that can be reduced and compensated elsewhere, such that the degree of target confidence can be maintained, based on a hypothetical spherical model with ideally conformal dose distributions.

In this paper, we present a technique to generate beam-dependent PTVs (bdPTVs) that are adapted away from specified OARs, building upon the work by Tsang *et al* (2017). Doing so allows the lowering of high-dose to regions where a traditional PTV overlaps with an OAR, such that better high-dose-sparing can be achieved for the OARs under consideration. Formally, the key requirement of adequate target coverage, expressed as a condition over the probability density function of uncertainties, is not changed. As such, the required level of dose coverage, on a beam-by-beam basis, is maintained following the same mathematical foundations as used by van Herk’s margin recipe (Van Herk *et al* 2000). The cost of the benefit to better spare an OAR is the irradiation of larger healthy tissue volumes, which are not classified as important organs at risk.

We present an example for the introduction of this concept using the prostate cases. A planning study is used to compare the dosimetric effects between using bdPTVs without adapting away for any OARs, adapting the bdPTVs to better spare the rectum only, and adapting the bdPTVs away from both the rectum and bladder, with a higher priority on the rectum.

## 2. Method

This paper formalises the generation of adapted beam-dependent PTVs, and is based upon the margin concept presented in Tsang *et al* (2017). The methodology outlined in this work is implemented into our in-house planning system DynaPlan (Kamerling *et al* 2016) and treatment planning optimiser *μ*Konrad (Ziegenhein *et al* 2013). The optimisation framework for use with beam-dependent PTVs and the methodology pertaining to plan evaluation and comparison follows from Tsang *et al* (2017) and remains unchanged in this work.

### 2.1. Generating adapted beam-dependent PTVs

In our approach, the PTV is generated by morphologically dilating the CTV using a margin mask; this is implemented by centring the margin mask at every voxel within the CTV, and the union of these masks constitutes the PTV. The beam-dependent margin concept proposed by Tsang *et al* (2017) considers uncertainties only in the directions perpendicular to beam direction. The corresponding margin mask takes the form of an ellipse, with the length of the semi-major/minor axes $\boldsymbol{M}$ defined by (1). Variables $\boldsymbol{\Sigma}$, $\boldsymbol{\boldsymbol{\sigma}_r}$ and $\boldsymbol{\boldsymbol{\sigma}_p}$ are two-dimensional column vectors for the directions perpendicular to the incident beam angle, representing the systematic uncertainties, random uncertainties and the penumbra, defined as the distance between the 20% and 80% isodose levels, respectively. The operations $\boldsymbol{x}^{\circ 2}$ and $\boldsymbol{x}^{\circ \frac{1}{2}}$ are the Hadamard square and the Hadamard root respectively.

1 \begin{align*} \newcommand{\e}{{\rm e}} \displaystyle \boldsymbol{M} = \alpha \boldsymbol{\Sigma} + \beta \left ( \left(\boldsymbol{\sigma}_r^{\circ 2} + \boldsymbol{\sigma}_p^{\circ 2}\right)^{\circ\frac{1}{2}} - \boldsymbol{\sigma}_p \right). \label{eq:vhmr} \nonumber \end{align*}

The coefficients *α* and *β* depend on the intended probability of target dose coverage. These are calculated by solving the closed-formed dose population histogram, following the integrals set out in appendix 2 of Van Herk *et al* (2000). For our case of 2D margins, to ensure that 90% of the patients receive at least 95% of the prescribed dose across the whole of the target, the corresponding coefficients are $\alpha=2.15$ and $\beta=1.64$.

In this work, the systematic and random uncertainties components of the margin mask are considered separately; in other words, instead of generating the PTV by morphologically dilating the CTV using one margin mask, this is separated into two steps: to morphologically dilate the CTV using a margin mask to account for the systematic uncertainties, and then dilating the resulting volume using a margin mask to account for the random uncertainties. Whilst these two methods result in theoretically identical PTVs, the latter method allows us to modify the margin mask for either the systematic/random uncertainties without affecting the other. In the rest of this paper, the term ‘margin mask’ refers solely to the one used to account for systematic uncertainties.

The margin needed to account for the systematic uncertainties is determined as the collection *U* of displacements $\boldsymbol{r}$ from the target’s mean position, such that (2) is satisfied, where *p*_*c*_ is the intended target coverage probability and $P(\boldsymbol{r})$ is the probability density distribution of the systematic uncertainties. For systematic uncertainties described by a (2D) Gaussian distribution, *U* ideally takes the form of an ellipse in order to minimise the area of the margin mask, with semi-major and -minor axes determined by 2.15$\boldsymbol{\Sigma}$ for *p*_*c*_  =  0.90.

2 \begin{align*} \newcommand{\e}{{\rm e}} \displaystyle p_c = \int_U P(\boldsymbol{r}) d\boldsymbol{r}. \label{eq:vhmr_der_int} \nonumber \end{align*}

**Figure 1.**
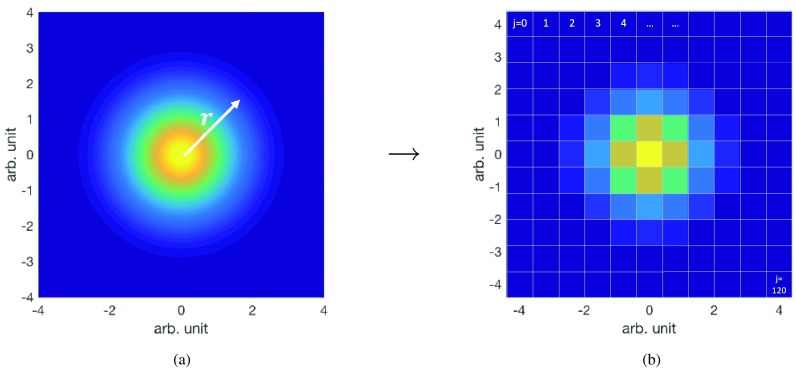
Moving from continous space of a Gaussian distribution of unit variance, with displacements $\boldsymbol{r}$ relative to the mean position centred at [0, 0], to discrete space where the elements are labelled by index *j*.

To cater for the voxelised nature of the patient scan, we move the margin concept from continuous space to discrete space (i.e. $\boldsymbol{r} \rightarrow j$), where *j* is the index of the elements on a discretised grid, as demonstrated in figure 1. The discretised version of (2) is given by (3), where the collection $V$ is made up of elements *j*, and the probability that the displacements $\boldsymbol{r}$, following P($\boldsymbol{r}$), lie within element *j* is given by *p*_*j*_; i.e. $p_j = \int\int_j P(\boldsymbol{r}) {\rm d} A$, where *A* is the area spanned by the element *j*. The values are then normalised such that $\sum_j p_j=1$, to remove any errors due to discretisation effects. The inequality in (3) is used to reflect the possibility that the cumulative probability of collection of voxels $V$ would not equate exactly to *p*_*c*_. Instead, a conservative inequality is used, where slight over-coverage of the tumour target is preferred over slight under-coverage, at the expense of slight increase in dose to surrounding OARs. The minimisation operation (${{\rm min}}$) is used to limit the equation to yield one solution with cumulative probability closest to the intended target coverage probability *p*_*c*_. To minimise the extent of the margin, an additional criterion is included: all elements *j* within the collection $V$ need to have a probability *p*_*j*_ larger than those outside the collection (i.e. the margin mask in the form of $V$ contains only elements *j* with the highest probabilities *p*_*j*_).

3 \begin{align*} \newcommand{\e}{{\rm e}} \displaystyle p_c \leqslant \min \left\{\sum_{j \in V} p_j : p_j &gt; p_{j'} \ \forall j \in V, {j'} \notin V \right\}. \label{eq:vhmr_der_num} \nonumber \end{align*}

For our adaptive margin formulation, a different collection *W* of elements *j* are selected, based on a weighted score given by (4) to satisfy (5). The variable *f*_*j*_ is a weighting factor to alter the priority of element *j* to be included as part of the margin mask. In other words, *w*_*j*_ governs the selection priority of element *j* that forms the margin mask, such that the cumulative probability covered by the elements $j \in W$ still equals to the required target coverage probability *p*_*c*_.

4 \begin{align*} \newcommand{\e}{{\rm e}} \displaystyle w_j = f_j \ p_j \label{eq:wj} \nonumber \end{align*}

5 \begin{align*} \newcommand{\e}{{\rm e}} \displaystyle p_c \leqslant \min \left\{\sum_{j \in W} p_j : w_j &gt; w_{j'} \ \forall j \in W, {j'} \notin W \right\}. \label{eq:adapt_num} \nonumber \end{align*}

The grids storing *f*_*j*_ and *w*_*j*_ are of same dimensions and level of discretisation as the grid storing *p*_*j*_, enabling the straightforward evaluation of *w*_*j*_. By modifying how *f*_*j*_ is defined, the formulation of the adaptation would vary.

The beam-dependent margin concept can be readily extended for use with arc therapy by discretising the arc into a finite number of control points and treating each control point as a separate beam for margin-generation purposes.

### 2.2. Formularising the adaptive weighting factor

For our adaptive beam-dependent margin approach, we define the weighting factor *f*_*j*_ following (6), and introduce two new parameters *ζ* and $\gamma_j$. *ζ* is a user-input variable, used to vary the degree of adaptation, and should only assume values larger than zero; $\zeta&lt;0$ would result in the margin adapting towards the OAR. Parameter $\gamma_j$ describes the proximity of an OAR relative to the CTV in the directions perpendicular to the beam axis.

6 \begin{align*} \newcommand{\e}{{\rm e}} \displaystyle f_j = \left( 1- \gamma_j\right)^\zeta. \label{eq:fj_clinical} \nonumber \end{align*}

**Figure 2.**
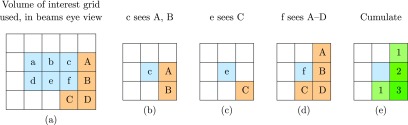
Example on how $\gamma_j$ is generated. (a) The volume of interest grid used for demonstration, with the CTV in blue, and OAR in orange. In this demonstration, we only consider ‘proximity’ as a chessboard distance of 1 relative to the CTV voxel; in practise, we consider displacements up to 3 standard deviations of the systematic uncertainty. ((b)–(d)) $\gamma_j$ for each of the CTV voxel with the OAR present within 1 chessboard distance. CTV voxels (a), (b) and (d) do not have any OAR voxels within 1 chessboard distance, and thus do not contribute to the $\gamma_j$’s overall score. (e) Accumulating all $\gamma_j$ scores. The final values used for weighting the distribution in (6) are obtained by dividing all values by the maximum value, scaling $\gamma_j$ to values between 0 and 1.

Figure 2 provides a graphical explanation on how $\gamma_j$ is generated. The $\gamma_j$ map takes the form of a grid at the same discretisation as the probability density map *p*_*j*_; in this example, the *p*_*j*_ map (not shown) and $\gamma_j$ map (derivative of figure 2(e)) take the form of $3\times3$ grids. This $\gamma_j$ map is centred on each voxel of the CTV, with the plane of the grid perpendicular to beam direction. The score of each element on the grid is increased if the grid element $\gamma_j$ is inside the contour of the OAR; only one OAR is considered, and all other OARs do not contribute to the score. All scores within the $\gamma_j $ map are then divided by the maximum score, scaling all scores to lie within the interval $\left[0, 1\right]$. As this process is repeated for all CTV voxels, the $\gamma_j$ map will include pseudo-depth information depending on the thickness of the CTV in beam direction.

For adapting bdPTVs away from multiple OARs, the weighting factor *f*_*j*_ is used for each OAR *m* considered, for a total number of *M* OARs, as described in (7); each OAR has its own $\gamma_j^m$ map and user-defined variable $\zeta_m$.

7 \begin{align*} \newcommand{\e}{{\rm e}} \displaystyle f_j &amp;= \prod_{m}^{M} f_j^m \nonumber \\ &amp;= \prod_{m}^{M} \left( 1- \gamma_j^m\right)^{\zeta_m} . \label{eq:fj_multi} \nonumber \end{align*}

### 2.3. Finding an optimal set of adapted bdPTVs

There is a correlation between a larger $\zeta_m$ and a larger overall volume of the bdPTVs. This is due to the shift from prioritising voxel with higher *p*_*j*_ to prioritising voxel with larger *w*_*j*_ when determining the margin mask, with a constraint of maintaining the same coverage at *p*_*c*_. A larger $\zeta_m$ also results in a smaller intersection volume between the OAR *m* and the bdPTVs, which is ideal for high-dose sparing of the OAR. This is because the bdPTVs corresponds to where dose is targeted to when solving for the inverse-planning optimisation problem. However, the resulting shape of the adapted margin masks, and consequentially the adapted bdPTVs, cannot be accurately determined *a priori*. This is due to the dependence of the adapted margin masks on input parameters $\zeta_m$, a user-defined parameter, and $\gamma_j$, which is dependent on the patient’s anatomical geometries. To determine the ‘ideal’ set of $\zeta_m$, an exhaustive set of bdPTVs needs to first be generated across a range of $\zeta_m$ for all OARs under consideration for adaptation, followed by an automated selection process.

The automatic selection process can be separated into four steps. First, bdPTVs for all possible combinations of $\zeta_m$ for all considered OARs, for a range of $\zeta_m = [0, 15]$, are generated to populate the solution space. All OARs not considered for margin adaptation are treated in the same manner as uncategorised healthy tissue. Second, an elimination step is used to remove any adapted bdPTVs from the solution space for cases where any of the considered OARs are ‘worse-off’ when compared to the set of bdPTVs where adaptation is not considered. The term ‘worse-off’ here is defined as the increase in volume of the intersection between the union of the bdPTVs ($ \newcommand{\bi}{\boldsymbol} \bigcup$bdPTVs) and the OAR. This is to ensure the resulting selected set of bdPTVs is always adapted away from all OARs under consideration. Third, the solutions are filtered by considering each OAR in order of its priorities, such that the volume of intersection between the $ \newcommand{\bi}{\boldsymbol} \bigcup$bdPTVs and the OAR under consideration is minimised. A tolerance of 1% relative to the minimum volume of the intersection is used, to retain more solutions to benefit subsequent steps of the selection process. For example, if the minimum volume of the intersection amongst the remaining solutions is 450 voxels, all solutions with a volume of intersection at 455 voxels or below are kept for further consideration (either for subsequent OARs, or for the fourth step of the selection algorithm). The fourth and final step is the overall minimisation for the volume of the $ \newcommand{\bi}{\boldsymbol} \bigcup$bdPTVs.

### 2.4. Planning study

The same five prostate patients from Tsang *et al* (2017) are used in this study. The dose-volume constraints following the PACE clinical trial recommendations (NCT01584258), as well as the dose prescription to the CTV and geometric uncertainties, are carried over into this study, with the target coverage assumed to be satisfied if at least 98% of the volume received at least 95% of the prescribed dose to 90% of the population.

A set of adapted bdPTVs is selected using the heuristic automated selection approach, as described in section 2.3. Three plans are generated for each patient: one using bdPTVs without adapting for any OARs, one with bdPTVs adapted away from the rectum, and one with bdPTVs adapted away from both the rectum and bladder, with the rectum taking a higher priority. For comparison purposes, the $\zeta_\mathrm{rec}$ used for adapting beam-dependent margins away from only the rectum are the same values as for adapting beam-dependent margins for both the rectum and bladder.

The optimisation objectives used are outlined in table 1, performed with direct aperture optimisation (Wild *et al* 2015) using 25 iterations for 40 segments. The objectives are not modified in this planning to better the plans, even if treatment plans can be improved, such that a systematic comparison between the plans can be conducted.

**Table 1.** IMRT inverse-planning optimisation objecitves used to generate the treatment plans used in the planning study. No changes to the objecitves are made to improve the treatment plans.

Organ	Function	Dose /Gy	Weight
Prostate (PTV)	Min dose	74.5	10
	Max dose	82.0	10
Rectum	Max dose	60.0	8
Bladder	Max dose	65.0	6
Femoral heads	Max dose	50.0	2
External	Max dose	60.0	2

For plan evaluation and comparison, a population size of 50 000 is used with the probabilistic evaluation tool described in Tsang *et al* (2017). The DVH criteria used for comparison are: 98% of the target volume ($D_{98\%}$) to receive at least 95% of the prescribed dose, as representative of dose coverage; the volume of rectum receiving 70 Gy and 75 Gy should not exceed 5% and 2%, respectively, as representative of high dose to the rectum; and the volume of bladder receiving 70 Gy and 74 Gy should not exceed 5% and 2% respectively, to be representative of high dose to the bladder. Dose-volume coverage maps (DVCMs) are generated for the rectum and bladder, again using a population size of 50 000. For comparison purposes, dose volume coverage difference maps (DVCDMs) are generated by subtracting the values of the DVCM for one plan from another.

## 3. Results

### 3.1. Generating adapted beam-dependent PTVs

**Figure 3.**
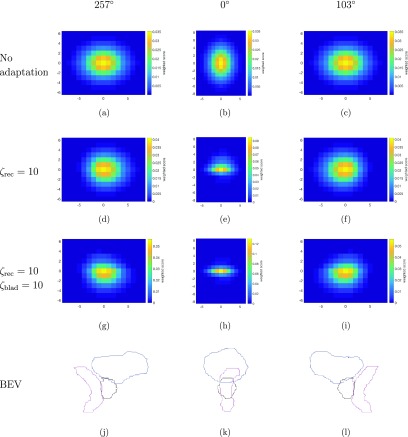
((a)–(i)) *w*_*j*_ maps for beams at gantry angles 257°, 0° and 103°, for generating margins without adapting for any OARs, adapting for the rectum with $\zeta_\mathrm{rec}=10$, and adapting for both the rectum and bladder with $\zeta_m=10$ for both OARs. The *x* and *y* axes represent the displacement from the centre of the margin mask in number of voxels. ((j)–(l)) Beam’s eye view (BEV) projections for the same beam angles, showing the contours of the prostate (CTV) in black, rectum in magenta, and bladder in blue.

Figures 3(a)–(i) show the *w*_*j*_ maps without adaptation, adapting for the rectum with $\zeta_\mathrm{rec}=10$ and adapting for both the rectum and bladder with $\zeta_\mathrm{rec}=10$ and $\zeta_\mathrm{blad} =10$ for beams at gantry angles 257°, 0° and 103°; the *w*_*j*_ maps are equivalent to the *p*_*j*_ maps in the case where adaptation is not considered. Figures 3(j)–(k) are used to show the projected contours of the prostate, rectum and bladder in beam’s eye view (BEV) for the same beams at gantry angles 257°, 0° and 103°.

**Figure 4.**
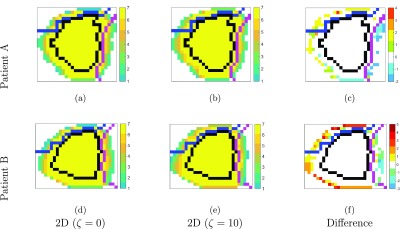
Isocenter sagittal slices for two different patients, ((a)–(c)) and ((d)–(f)), showing the degree of overlap between all beam-dependent PTVs, for ((a), (d)) no adaptation and ((b), (e)) with adaptation for the rectum using $\zeta = 10$. The values shown are before being scaled to values between 0 and 1 for ease of readability. The CTV is outlined in black, the rectum in magenta, and bladder in blue. ((c), (f)) The difference maps between the corresponding overlap map without and with adapatation. Positive values indicate the voxel contributing to more bdPTVs using the adapted margins, and vice-versa for negative values.

Figures 4(a) and (b) show the isocenter sagittal slices of the degree of overlap between all bdPTVs (i.e. these are the $o_k^i$ maps used for optimisation, please refer to section 2.2 of Tsang *et al* (2017) for a detailed explanation) for one prostate case, using a $\zeta_\mathrm{rec}$ value of 0 (i.e. without adaptation) and 10 for the rectum, respectively, to demonstrate how adapted bdPTVs differs to unadapted bdPTVs. Comparing these two figures visually, it may be difficult to determine their differences. Figure 4(c) shows the differences in the degree of overlap by pixel-wise subtracting the values in figures 4(a) from (b). Figures 4(d)–(f) are isocenter sagittal slices for a different prostate case, showing the degree of overlap without adaptation, with adaptation for the rectum and differences respectively, to demonstrate how the patient’s anatomical geometry also plays a role in determining the degree of adaptation when using the same $\zeta_\mathrm{rec}$.

**Figure 5.**
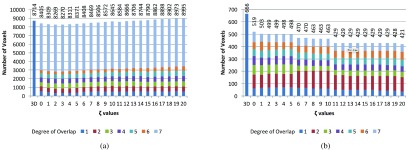
Stacked bar charts showing number of voxels for each degree of overlap between all beam-dependent PTVs across a range of *ζ* values, (a) for all voxels within the union of the bdPTVs ($ \newcommand{\bi}{\boldsymbol} \bigcup$bdPTVs) and (b) only for voxels in the intersection between the $ \newcommand{\bi}{\boldsymbol} \bigcup$bdPTVs and the rectum. The numbers at the top of each bar show the total number of voxels.

Figure 5(a) shows the how the degree of overlap varies, in terms of number of voxels, between all bdPTVs across a range of $\zeta_\mathrm{rec}$ when considering only the rectum for margin adaptation. Here, the number of voxels is used as a surrogate for volume, where each voxel has a volume of 9.2 mm^3^ for all patient cases. This is to demonstrate the relationship between the $\zeta_m$ used and the size and shape of the bdPTVs; for such demonstration, only one OAR is considered for simplicity. The number of voxels for the PTV generated using Van Herk’s (3D) margin recipe is also included for comparison. The majority of each column is the ‘full’ overlap (i.e. degree of overlap is 7, for 7 beams), as this volume includes the CTV itself. As the size of the (bd)PTVs usually correspond with the integral dose delivered to the patient, this should ideally be kept as low as possible.

Figure 5(b) considers only the voxels within the intersection between the rectum and the union of the bdPTVs ($ \newcommand{\bi}{\boldsymbol} \bigcup$bdPTVs), where a lower value implies better adaption of the bdPTVs away from the rectum. These two figures clearly demonstrate the trade-off between adapting bdPTVs away from the OAR and increasing the size of the bdPTVs as a result.

The relationship between the degree of overlap of the bdPTVs and the different $\zeta_m$ used when considering two or more OARs for margin adaptation is not as straightforward, owing to the relative position between the OARs and the target influencing the formation of the *w*_*j*_ maps. This is the primary reason why a heuristic selection process, such as the one proposed in section 2.3, is required to determine the set of adapted bdPTVs that should be used for treatment planning.

### 3.2. Finding an optimal set of adapted bdPTVs

**Figure 6.**
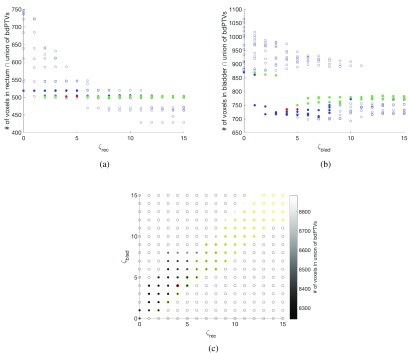
((a), (b)) Plot showing the relationship between *ζ* for the (a) rectum and (b) bladder, and number of voxels within the intersection between the OAR and the union of the beam-dependent PTVs ($ \newcommand{\bi}{\boldsymbol} \bigcup$bdPTVs). Empty markers represent combinations of *ζ* that are discarded due to the number of voxels in OAR $ \newcommand{\bi}{\boldsymbol} \cap \ \left(\bigcup \mathrm{bdPTV}\right)$, for any OAR under consideration, to be larger than that of the scenario where no adaptations are considered. Solid blue markers are all remaining combinations of *ζ* under consideration, green markers are remaining margins after considering minimising the number of voxels within rectum $ \newcommand{\bi}{\boldsymbol} \cap \ \left(\bigcup \mathrm{bdPTV}\right)$, red markers are margins that are remaining after considering for the bladder as well. (c) Plot showing the how $\zeta_\mathrm{rectum}$ and $\zeta_\mathrm{bladder}$ affect the overall volume of the $ \newcommand{\bi}{\boldsymbol} \bigcup$bdPTVs (i.e. inclusive of all voxels belonging to one or more beam-dependent margins), represented using a grayscale. Empty markers represent plans that are not considered as they are not improvements over unadapted plans for at least one of the considered OARs. Yellow markers represent combinations of *ζ* with the smallest intersection between the rectum and the $ \newcommand{\bi}{\boldsymbol} \bigcup$bdPTVs, within tolerance; green markers represent the combinations of *ζ* that are preferential for both rectum and bladder. The red marker represents the combination of *ζ* that is used to generate the bdPTVs used for treatment planning for this patient.

Figure 6 presents diagrammatically the elimination and selection process for choosing the adapted bdPTVs to be used for treatment planning for one prostate case, following the framework laid out in section 2.3. Solutions that result in an increase in volume of the intersection between the $ \newcommand{\bi}{\boldsymbol} \bigcup$bdPTVs and any of the OARs, relative to the set of bdPTVs where adaptation is not considered, are first removed. The volume of the intersection between the $ \newcommand{\bi}{\boldsymbol} \bigcup$bdPTVs and the rectum is then minimised, followed by the consideration for the bladder. Lastly, the solution with the smallest volume of the $ \newcommand{\bi}{\boldsymbol} \bigcup$bdPTVs is selected.

Table 2 shows the $\zeta_m$ representing the adapted bdPTVs chosen by the selection process, the number of voxels within the intersection between the OAR and the $ \newcommand{\bi}{\boldsymbol} \bigcup$bdPTVs, and the degree of improvement relative to the set of bdPTVs without adaptation considered.

**Table 2.** Parameters $\zeta_\mathrm{rec}$ and $\zeta_\mathrm{blad}$ associated with the adapted bdPTVs used for the planning study, following the framework laid out in section 2.3. The number of voxels within the intersection between the rectum/bladder and the union of the bdPTVs are shown, and compared to their respective unadapted bdPTVs.

Patient	Rectum $\cap$ $ \newcommand{\bi}{\boldsymbol} \bigcup$bdPTVs			Bladder $\cap$ $ \newcommand{\bi}{\boldsymbol} \bigcup$bdPTVs		
	$\zeta_\mathrm{rec}$	# of voxels	Diff from $\zeta=0$	$\zeta_\mathrm{blad}$	# of voxels	Diff from $\zeta=0$
1	10	536	−80	6	964	−86
2	6	569	−99	4	1821	−295
3	4	503	−16	4	727	−143
4	13	495	−68	7	1346	−70
5	14	266	−6	4	805	−210

### 3.3. Planning study

Table 3 presents the target coverage probabilities for the five patients comparing between plans without adapting for any OARs, adapting for the rectum only using $\zeta_\mathrm{rec}$ in the first column of table 2, and adapting for both the rectum and bladder using $\zeta_m$ in the same table. All plans were able to satisfy the required target coverage probability of 90%.

**Table 3.** Results from the verification tool, using a population of 50 000. The values show the probabilities, in percentages, where the minimum dose to 98% of the CTV’s volume ($D_{98\%}$) is at least 95% of the prescribed dose, to two decimal places. The values here compare plans generated using beam-dependent PTVs without being adapted for any OARs (std), adapted for the rectum only (rec) and adapted for both the rectum and bladder (r&b).

	CTV: $D_{98\%} &gt; 95\% \ D_\mathrm{pres}$		
Patient	2D (std)	2D (rec)	2D (r&b)
1	96.50	95.52	96.16
2	99.68	99.82	99.90
3	94.96	94.22	94.47
4	99.68	98.60	93.04
5	99.48	99.18	99.18

**Figure 7.**
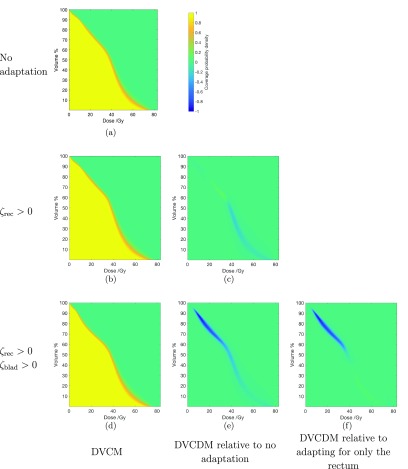
((a), (b), (d)) Dose volume coverage maps (DVCMs) for the rectum of one patient, generated using a population of 50 000, for treatment plans using beam-dependent PTVs without being adapted for any OARs, adapted for only the rectum, and adapted for both the rectum and bladder, respectively. The colourbar next to (a) is used for all subfigures, and represents the probability that the dose observed for a given volume in an individual treatment course is equal to, or higher than, the specified point on the DVCM. Negative values are only used in dose volume coverage difference maps. ((c), (e), (f)) Dose volume coverage difference maps (DVCDMs) showing the differences between the DVCMs.

**Figure 8.**
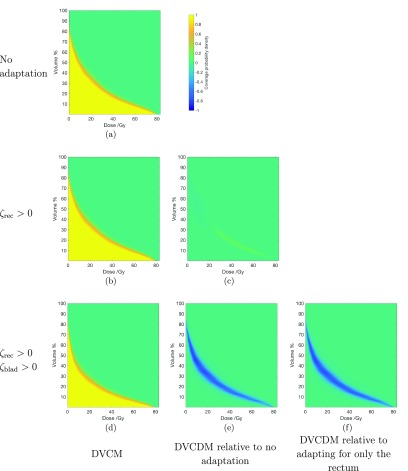
((a), (b), (d)) Dose volume coverage maps (DVCMs) for the bladder of one patient, generated using a population of 50 000, for treatment plans using beam-dependent PTVs without being adapted for any OARs, adapted for only the rectum, and adapted for both the rectum and bladder, respectively. The colourbar next to (a) is used for all subfigures, and represents the probability that the dose observed for a given volume in an individual treatment course is equal to, or higher than, the specified point on the DVCM. Negative values are only used in dose volume coverage difference maps. ((c), (e), (f)) Dose volume coverage difference maps (DVCDMs) showing the differences between the DVCMs.

Table 4 presents the probability of the volume of rectum receiving 70 Gy and 75 Gy (i.e. $V_\mathrm{70~Gy}$ and $V_\mathrm{75~Gy}$) to not exceed 5% and 2%, and table 5 presents the probability of the bladder receiving $V_\mathrm{70~Gy}$ and $V_\mathrm{74~Gy}$ to not exceed 5% and 2%. Figures 7 and 8 presents the DVCM and DVCDMs for the rectum and bladder across the three plans for one of the patients.

**Table 4.** Results from the verification tool, using a population of 50 000. The values show the probabilities, in percentages, that the DVH objectives for the rectum are satisfied, to two decimal places. The values here compare plans generated using beam-dependent PTVs without being adapted for any OARs (std), adapted for the rectum only (rec) and adapted for both the rectum and bladder (r&b).

	Rectum: $V_{70~\mathrm{Gy}} &lt; 5\%$			Rectum: $V_{75~\mathrm{Gy}} &lt; 2\%$		
Patient	2D (std)	2D (rec)	2D (r&b)	2D (std)	2D (rec)	2D (r&b)
1	74.56	84.12	83.20	85.06	86.52	86.12
2	62.42	80.16	76.12	47.96	67.44	62.30
3	78.60	82.05	80.28	89.55	90.84	89.25
4	24.92	41.24	41.42	29.32	56.92	56.42
5	86.92	91.82	90.26	92.32	95.70	95.44

**Table 5.** Results from the verification tool, using a population of 50 000. The values show the probabilities, in percentages, that the DVH objectives for the bladder are satisfied, to two decimal places. The values here compare plans generated using beam-dependent PTVs without being adapted for any OARs (std), adapted for the rectum only (rec) and adapted for both the rectum and bladder (r&b).

	Bladder: $V_{70~\mathrm{Gy}} &lt; 5\%$			Bladder: $V_{74~\mathrm{Gy}} &lt; 2\%$		
Patient	2D (std)	2D (rec)	2D (r&b)	2D (std)	2D (rec)	2D (r&b)
1	96.90	90.22	98.06	57.92	46.50	70.14
2	73.34	58.46	87.64	11.42	6.88	22.06
3	99.99	99.99	99.99	94.13	92.73	98.84
4	99.99	99.94	99.99	89.72	79.34	93.04
5	50.12	42.66	86.20	12.52	10.80	45.06

These results show consistently that any adaptation is better than no adaptation. First, when considering the adaptation of the bdPTVs for only the rectum, the bladder tends to receive additional high dose. This is the consequence of the generation of adaptive margin aiming to minimise the overall volume of the bdPTVs. When the bladder is also considered for adaptation, a reduction of high-dose sparing for the rectum can be observed, though results are still better than if no adaptations were performed. This is due to the different elements *j* used to define the margin mask, following (5) and based on the variable *w*_*j*_ determined by (7), used to expand the CTV into the bdPTVs for each beam direction.

## 4. Discussion

Our adaptive margin concept models the overall effect of random uncertainties as blurring of the cumulative dose distribution, similar to many margin-based and margin-less approaches used to account for geometric uncertainties in radiotherapy within the literature. However, doing so assumes the treatment to be delivered over a large number of fractions, and is unsuitable for use with hypo-fractionated treatment schemes for mainly two reasons: one, the averaging of the random uncertainties would no longer be zero, i.e. there exist residual systematic uncertainties that would also need to be accounted for (Van Herk *et al* 2000); and two, the margin recipe fails to consider the consequence of the non-negligible dose variability due to random uncertainties, i.e. the assumption that the random uncertainties can be modelled as a blurring of the cumulative dose distribution breaks down (Gordon and Siebers 2007).

The BEV projections shown in figures 3(j)–(l) shows the outline of the VOI in beam direction, and therefore does not contain any depth information, i.e. does not show the placement of the VOIs along the beam axis. These projections therefore will not portray how two VOIs are placed with respect to one another, in beam direction, which may affect the formation of the $\gamma_j$ maps, and subsequently the *w*_*j*_ maps. These BEV projections still serve as a valuable tool in providing the confirmation that the *w*_*j*_ maps are weighted in accordance to the relative placements of the VOIs in the directions perpendicular to the beam axis. Figure 3(e) provides an example where the position of the OAR, the rectum shown in magenta, relative to the CTV, shown in black, in beam direction is not immediately apparent. The rectum curves away from the prostate superiorly and posteriorly, therefore adaptation in the superior direction is less critical compared to the inferior part of the CTV, for example. This is reflected in the *w*_*j*_ map when considering only the rectum for adaptation of the bdPTVs. All other *w*_*j*_ maps behave as expected, where a lower magnitude is observed when compared to the *p*_*j*_ maps in the directions where an OAR under consideration is present.

Figure 5 confirms the two assumption that are made in the automated selection process outlined in section 2.3. Figure 5(a) confirms the correlation between a larger $\zeta_m$ used and the overall extent of the bdPTVs, and figure 5(b) confirms a higher $\zeta_m$ used correlate to a reduction in overlap between the bdPTVs and the OAR *m* that is being considered. Figure 5 also demonstrates the effects of discretisation on how the margins, and by extension the bdPTVs, are generated. Step changes in the number of voxels within the union of the bdPTVs at particular values of *ζ* are observed in figure 5(b), in contrast to the more gradual increase observed in figure 5(a). This can be attributed to the discretisation of the $\gamma_j$, *p*_*j*_ and *w*_*j*_ grids used, which the margin mask is dependent on for its generation. As the margin mask is used to morphologically dilate the CTV to form the bdPTVs, even a difference of one voxel in the margin mask can have an observable effect on the resulting bdPTVs.

The automated selection of the adapted bdPTVs provides a systematic, hierarchical approach to minimising the volume of intersection between the adapted bdPTVs and the multiple OARs under consideration. The initial step used to remove all combinations of $\zeta_m$ that result in larger intersections between the $ \newcommand{\bi}{\boldsymbol} \bigcup$bdPTVs and any OAR considered for adaptation, relative to the set of bdPTVs where adaptation is not considered, is necessary due to the competing nature between OARs within the adaptive margin concept. Without this second step of the selection process, the minimisation of the intersection volume between the $ \newcommand{\bi}{\boldsymbol} \bigcup$bdPTVs and the first OAR would limit the solution space for all subsequent OARs, possibly containing only solutions where the volumes of intersection between the $ \newcommand{\bi}{\boldsymbol} \bigcup$bdPTVs and the OARs with lower priorities are larger than for the case where adaptation is not considered. There is also a risk of the framework eliminating all solutions if a large number of OARs are considered for adaptation. This is due to the need for each of the bdPTV to be of a certain minimum size to ensure the required probability of target coverage, and the OARs will inevitably be competing against one another. The second step of the selection algorithm should therefore entertain the possibility of relaxing the strict elimination criteria when adapting for a large number of OARs. This study was restricted to the use of two OARs, and the robustness of this method relative to the number of OARs under consideration for adaptation could not be ascertained.

The third step of the selection process permits a small tolerance in the minimisation process, allowing lower $\zeta_m$ values to be chosen. This is intended to retain more solutions for subsequent considerations for OARs with lower priorities, such that more favourable results, in the form of a smaller intersection volumes between these subsequent OARs and the bdPTVs, are possible. Moreover, this tolerance also encourages the possible reduction in the overall extent of the bdPTVs by having more solutions to consider during the fourth step of the selection process.

The planning study conducted in this investigation uses the same patient cases in Tsang *et al* (2017) for consistency. All plans share the same optimisation objectives in this investigation to allow for a more systematic comparison on how adapted beam-dependent margins affect the dose distribution, based on results from the DVCMs. Otherwise, it would be more difficult to differentiate changes in the dose distribution due to changes in the optimisation objectives and changes in the bdPTVs. The effects of varying the optimisation objectives for a fixed set of bdPTVs have already been investigated in Tsang *et al* (2017).

From the target coverage probabilities seen in table 3, the use of stricter optimisation objectives to further improve OAR sparing is possible without violating the required target coverage probability of 90%. The trade-off in dose to the OAR would also be affected by the adaptive margin concept due to the additional trade-offs in the degree of overlap between the bdPTVs and OARs. The search for an ‘optimal’ set of optimisation objectives automatically to further improve OAR dose sparing without manual trial and error is outside the scope of this investigation.

This planning study limits the number of OARs under consideration to only two, and only demonstrates the use of adaptive margins to the prostate indication. The method can be readily applied to other anatomical sites, as the quantification of the proximity between the VOIs (for determining $\gamma_j$) is independent of the orientation and size of the VOIs. In this work, parameter $\zeta_m$ has been investigated between 0 and 15 when generating adapted bdPTVs to consider the presence of both the rectum and bladder. For other indications, the range of $\zeta_m$ considered for generating adapted bdPTVs may differ to the $\zeta_m$ considered in this planning study for the prostate indication. Regardless, $\zeta_m$ should always remain positive to adapt margins away from the OAR, as $\zeta_m$ below zero would result in the adapting of the margins towards the OAR. Moreover, modifications to the automated selection process described in section 2.3, in particular the second step of the selection process, may be required for other indications, from a binary decision in narrowing down the solution space to determining the trade-offs based on scoring of the solutions using an alternative criterion. Nonetheless, we have demonstrated the adaptive margin concept and the associated selection process presented in this work to be effective for the prostate indication in reducing the amount of high-dose delivered to both the rectum and bladder whilst achieving the clinically-required level of target coverage probabilities.

## 5. Conclusion

We have demonstrated the ability to spare high dose to one or more OARs by adapting the bdPTVs accordingly, at the expense of having larger bdPTVs and thus delivering more radiation to uncategorised healthy tissue or OARs that were not considered for adaptation. This enables us to maintain similar levels of target coverage probability relative to using unadapted bdPTVs for treatment planning.

The effectiveness of the adaptive margin concept depends on both the patient’s anatomical geometry and the $\zeta_m$ used to define the severity of the adaptation for OAR *m*. A process has been proposed to select the set of adapted beam-dependent PTVs for use in treatment planning, eliminating the need to manually choose a set of adapted bdPTVs, out of a large pool of viable solutions with varying degree of trade-offs.

From the accompanying planning study, we observe better OAR dose sparing using adapted bdPTVs compared to their unadapted counterparts. Dose trade-offs between the various volume of interests depend on the number of OARs considered for adaptation, the respective $\zeta_m$ used, and the geometry of the patient anatomy.
